# Smart edible films based on mucilage of *lallemantia iberica* seed incorporated with curcumin for freshness monitoring

**DOI:** 10.1002/fsn3.2114

**Published:** 2021-01-07

**Authors:** Pouya Taghinia, Anna Abdolshahi, Sahebeh Sedaghati, Behdad Shokrollahi

**Affiliations:** ^1^ Department of Food Science and Technology Islamic Azad University Sari Iran; ^2^ Food safety Research Center (salt) Semnan University of Medical Sciences Semnan Iran; ^3^ Department of Food Science and Technology Ferdowsi University of Mashhad (FUM) Mashhad Iran

**Keywords:** curcumin, food spoilage, smart packaging system, *Lallemantia iberica* seed gum

## Abstract

The objective of the present work was first to develop a smart packaging system based on *Lallemantia iberica* seed gum (LISG)/curcumin and, subsequently, investigate its physicochemical characteristics and biological activity. Finally, the response of LISG/curcumin films against pH change and the spoilage of shrimp were tested. The barrier properties and mechanical performance of the films improved as the curcumin concentration increased. FT‐IR analysis revealed the formation of physical interaction between LISG and curcumin. LISG/curcumin films showed a continuous and steady release of curcumin. The incorporation of curcumin into LISG matrix imparts antioxidant and antibacterial/mold activity to the films. A strong positive correlation was observed between total volatile base nitrogen (TVBN) content of shrimp and a* (redness) during storage time (Pearson correlation = 0.975). Eventually, LISG/curcumin film could be a promising smart packaging system capable of detecting food spoilage.

## Graphical abstract

Lallemantia iberica seed gum (LISG)/curcumin film is an active and intelligent packaging system that could be used to inhibit the microbial growth. Furthermore, it can be applied to detect the change in the quality of sea foods.

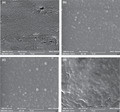

## 1 INTRODUCTION

Due to the ever‐increasing demand to develop smart packaging system capable of detecting food spoilage, the food sector is searching for new developments. The incorporation of pH‐sensitive compounds into packaging systems is one of the most attractive methods to fabricate packaging system with the capability of spoilage detecting (Aliabbasi et al., 2020). Curcumin is a bioactive compound extracted from the ground rhizomes of *Curcuma longa* L. This compound has diverse biological activities including antimicrobial, antioxidant, and anti‐HIV activities (Robles‐Almazan et al., 2017; Solghi et al., 2020). From a structural point of view, curcumin is a di‐phenolic substance with three labile hydrogen atoms. As the pH changes from acid to basic, the phenolic hydroxyl groups of curcumin react with OH and form phenoxide anion, correspondingly, the color of curcumin shifts from yellow to red. This property makes it appropriate for use in smart and intelligent packaging systems. Various studies have been carried out on the development of smart packaging systems incorporated by curcumin (Bajpai et al., 2015; Govindaraj et al., 2014; Mayet et al., 2014; Roy & Rhim, 2020; Wang et al., 2019).

The use of natural polymers to produce packaging systems with competitive mechanical performance as well as good physical and functional properties has attracted the attention of scientists in recent years. Proteins and polysaccharides are two main polymers that are extensively employed to produce sustainable films and packages (Galus & Lenart, 2013; Zolfi et al., 2014). *Lallemantia iberica* seed gum (LISG), as a biological macromolecule, is a galactomannan polysaccharide composed of galactose, mannose, rhamnose, and uronic acid (Fathi et al., 2018). Sadeghi‐Varkani et al. (2018b) optimized the formulation of biodegradable films based on LISG. The optimal formulation of LISG‐based film was found to be the biopolymer concentration of 1.2% and glycerol concentration of 35%. In this condition, the fabricated films had highest barrier and mechanical performance. In another study, Sadeghi‐Varkani et al. (2018a) developed a nanocomposite film based on LISG matrix incorporated by TiO_2_ nanoparticles. The authors observed that LISG nanocomposite had excellent physico‐mechanical characteristics and thus could be used in packaging applications to extend the shelf life of foods.

In the present work, for the first time, a smart packaging system formulated by LISG/curcumin was developed and its physicochemical and biological properties were investigated. Finally, the capability of the developed packaging system to monitor the freshness of shrimp was tested.

## 2 MATERIALS AND METHODS

### 2.1 Materials

*Lallemantia iberica* seeds were purchased from a local market in Sari, Iran. The extraction of mucilage from the seeds was carried out as described by Fathi et al. (2018). All the chemicals were obtained from Sigma‐Aldrich. Microbial cultures were supplied from Himedia.

### 2.2 Methods

#### 2.2.1 Smart film preparation

A dispersion containing 1% (w/v) *Lallemantia iberica* seed gum (LISG) in deionized water was prepared on a magnetic stirrer at ambient temperature and stored for 12 hr at refrigerator to be hydrated completely. An ethanolic solution of curcumin (0.2%–0.6% w/v) was also prepared, followed by the addition of tween 80 (150 mg ml^‐1^), as emulsifier under stirring. Afterward, the curcumin dispersion was added dropwise to the LISG dispersion with rate of 1 ml min^‐1^. Then, in order to evaporate ethanol from the dispersion, rotary evaporation (at 40ºC under vacuum condition) was carried out. Finally, the resulting dispersion was casted on teflonated Petri dishes (15 cm diameters), dried in an air dryer at 25°C for about 16 hr, peeled and stored in desiccator for further study.

#### 2.2.2 Morphological properties

Field emission scanning electron microscopy (FESEM) was employed to elucidate the morphology of LISG film with and without curcumin. First, the film samples were coated with gold and then their morphological properties were investigated using FESEM (Tescan, JMIRA3 LM). The instrument was operated at an accelerating voltage of 4 kV.

#### 2.2.3 Physical characteristics

##### Films thickness

The films’ thickness was measured by a hand‐held digital micrometer. Five film positions were selected to determine the thickness of the film. The mechanical and barrier properties of the films were computed using the average thickness.

##### Water solubility (WS), moisture content (MC), and water vapor permeability (WVP)

WS of the films was determined as reported by Gontard et al. (1994) with slight modifications. Briefly, the pieces of the film (2.0 × 2.0 cm^2^) were weighted, and dissolved in 50 ml of distilled water at 25ºC for 24 hr using an incubator equipped with a heating unit. Finally, the remaining pieces were filtered through Whatman nº 1 filter paper and dried at 105°C until reaching a constant weight (*W*1). WS was determined using the following equation:

(1) $$WS\left(\%\right)=\frac{W_{0}‐W_{1}}{W_{0}}\times 100$$

in which, $W_{0}$ indicates the initial dry weight.

In order to measure MC of the pieces of the film, first they were conditioned, and then their weight loss after heating at 105°C until reaching constant weight was recorded. MC value was calculated as follows (Zolfi et al., 2014):

(2) $$MC=\frac{\mathrm{Water weight loss}}{\mathrm{Moist film weight}}\times 100$$

Water vapor permeability of the films were determined as follows: The films were sealed over beakers containing silica gel (0% RH). Initially, the beakers were weighed every 9 hr and then weighted every 24 hr for 4 days. Following formula was used to calculate WVP of the films:

(3) $$WVP=\frac{\Delta m}{A\Delta t}\frac{X}{\Delta p}$$

here, Δm/Δt is weight gain per unit of time (g s^‐1^), X shows the films’ thickness (mm), and A is exposed the area of the films (m^2^) (ASTM Standard 1989).

#### 2.2.4 Mechanical properties

Mechanical performance of the developed films was evaluated using M350‐10CT Machine (Testometric Co.) as described by ASTM standard assay of D882‐91 (ASTM, 1991). The initial grip separation and crosshead speed were set at 50 mm and 50 mm min^‐1^, respectively. All the tests were carried out in five times. Mechanical parameters including tensile strength (TS) and elongation at break (EB) were determined from the stress–strain curve.

#### 2.2.5 FT‐IR analysis

The FT‐IR analysis was performed to investigate the chemical structures of LISG, curcumin, and LISG/curcumin films. 5 mg samples were grounded and pressed into a pellet with potassium bromide powder. The scanning was conducted in the wavenumber range of 550–4000 cm^‐1^.

#### 2.2.6 Release rate

The release rate of curcumin from LISG/curcumin film was determined as described by Kang et al. (2018). The films were cut with dye cutting press (2 × 2 cm) and then immersed in ethanol solution (5 ml). 2.5 ml of the solution was taken at various time intervals (0.5, 1, 3, 6, 9, 12, and 15 hr) and the absorbance was read at 428 nm using a UV‐vis spectrophotometer (Agilent Cary 100 Series). A standard curve was plotted, and the curcumin concentration in ethanolic solution was calculated according to the standard curve.

Different models, including zero‐order diffusion, Higuchi's diffusion, and Korsmeyer's Peppas models, were employed to describe the release profile of curcumin.

##### Antimicrobial and antimold activity

The antibacterial and antimold activities of the films were tested using zone inhibition method. The test was conducted against Escherichia *coli* O157:H7EDL 933, *Bacillus cereus* PTCC 1247, *Bacillus subtilis* PTCC 1023 *(*ATCC 6633), and *Penicillium expansum*. For this purpose, the film pieces (10 × 10 mm) were placed on solid nutrient agar inoculated by the tested bacterium, followed by incubating at 37°C for 2 days. The diameter of inhibition zone of the disk was recorded as the antibacterial potential of the samples.

##### DPPH radical scavenging activity

DPPH activity of the samples was tested according to the earlier described assay (Lai et al., 2010) with slight modifications. The films were immersed for 5 days, and then 2 ml of the resulting solution mixed with 2 ml of methanolic solution of DPPH (150 μM). The mixture was shacked vigorously, followed by keeping in the dark for 30 min at 37ºC. Finally, the absorbance was recorded at 517 nm using a UV‐vis spectrophotometer (Agilent Cary 100 Series). The scavenging effect was calculated as follows:

(4) $$\text{Scavenging}\;\text{activity(\%)}\;=\;\left[\frac{A_{517\text{of}\;\text{the}\;\;\text{control}}‐A_{517\text{of}\;\text{the}\;\;\text{sample}}}{A_{517\text{of}\;\text{the}\;\;\text{control}}}\right]\times 100.$$

##### Films response to pH changes

The color change of the smart films in facing the media with different pHs (3, 7 and 11) was recorded using a digital camera (Kodak M853). The color parameters including lightness (L*), redness (a*), and yellowness (b*) were measured using a chroma‐meter (Minolta Co. Ltd.).

###### Application of the films as an indicator of freshness for shrimp

LISG/curcumin film was used to detect the freshness of shrimp over storage time. 6 ± 0.3 g shrimps were weighted and covered with developed films. The covered shrimps were packaged by poly ethylene film and stored in an incubator at 25ºC and 75% RH. The values of total volatile basic nitrogen (TVBN) of the shrimps were measured over storage time by a stream distillation method as reported by Cai et al. (2011).

### 2.3 Statistics

SSPS software (Version 16) was used to analyze the experimental data. In order to compare the mean values, Duncan test was used.

## 3 RESULTS AND DISCUSSION

### 3.1 Physico‐mechanical and morphological properties of LISG‐based films incorporated by curcumin

#### 3.1.1 Film microstructure

**FIGURE 1:**
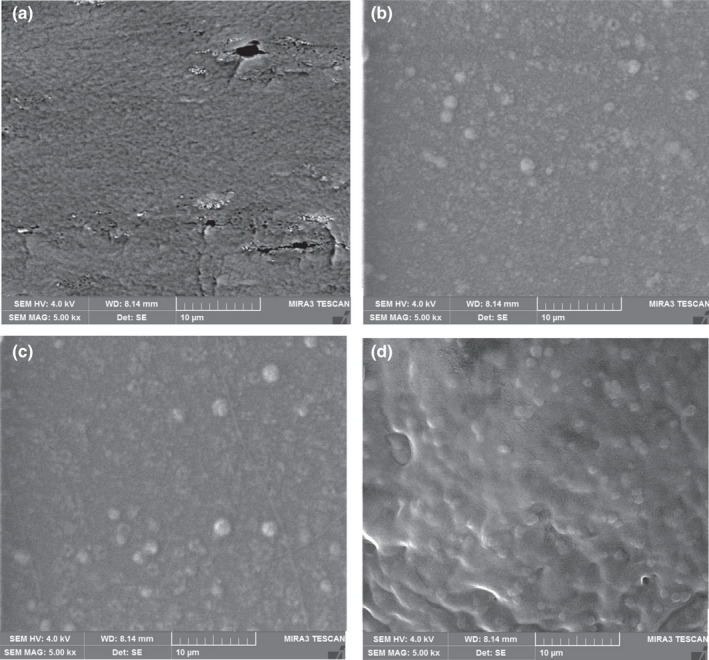
*SEM* micrographs of surface from LISG films incorporated with curcumin. (a) LISG film. (b) LISG‐CUR film incorporated with 0.2% curcumin, (c) 0.4% curcumin, and (d) 0.6% curcumin

The physical and mechanical properties of films are mainly dependent on interfacial adhesion between filler phase and polymer matrix as well as the microstructure of films (Fabra et al., 2011). In the present study, the microstructure of the neat films and those incorporated by different concentrations of curcumin were evaluated by FESEM analysis (Figure 1). It can be observed that in the neat film, there are some cracks and cavities that can increase the gas permeability of the films. However, in the films incorporated by curcumin especially those containing 0.6% curcumin, the entire surface of the films covered by micrometric curcumin crystals which can act as an obstacle for the diffusion of gasses. Moreover, addition of 0.6% curcumin resulted in a denser structure of LISG film. Thus, it is expected that LISG/curcumin (0.6%) has the lowest water vapor permeability and most mechanical performance which will be evaluated in the following sections. This observation is due to good compatibility between curcumin and LISG and filling of interspace of the films by curcumin microcrystals.

#### 3.1.2 Physical properties

The moisture content of the films is a crucial factor that affects the stability of the films and their brittleness. Furthermore, low content of moisture leads to the protection of films against physical and microbial spoilage (Thakur et al., 2016). The moisture content of LISG‐based film as a function of curcumin concentration is given in Table 1. When the curcumin concentration increased from 0% to 0.6%, the MC decreased significantly. This observation is related to the hydrophobic nature of curcumin (Rostami & Esfahani, 2019). This trend is in agreement those reported by Rostami and Esfahani (2019) who investigated the effect of curcumin incorporation on moisture content of *Melissa officinalis* seed gum‐based films. On the other hand, Liu et al. (2016) exhibited that the addition of curcumin had no considerable impact on MC of chitosan films.

**TABLE 1:** Physical characteristics of LISG films incorporated with various concentrations of curcumin

Curcumin conc (%)	Moisture content (%)	Water solubility (%)	WVP (×10^−6^ g^−1^ s^−1^ pa^−1^)
0	15.3 ± 0.71^a^	26.25 ± 0.71^a^	7.33 ± 0.76^a^
0.2	11.7 ± 0.68^b^	19.18 ± 0.64^b^	6.18 ± 0.61^a^
0.4	10.12 ± 1.08^b^	16.14 ± 0.82^d^	4.53 ± 0.033^b^
0.6	7.46 ± 1.02^c^	18.87 ± 0.79^c^	5.19 ± 0.011^ab^

Different letters in the columns indicate a significant difference of P < 0.05.

WS of the films is a critical property in food packaging and commonly considered as an indicator of water tolerance (Abdollahi et al., 2013; Salarbashi et al., 2018). The films with a low level of WS can effectively protect foods against physical and microbial spoilage. On the other hand, high level of WS can be considered as a desirable property, as it imparts a high degree of biodegradability (Stuchell & Krochta, 1994). As presented in Table 1, with increasing curcumin concentration from 0% to 0.6%, WS of the films decreased significantly which has been attributed to the hydrophobic nature of curcumin (Rostami & Esfahani, 2019). Pereira and Andrade (2017) indicated that as the curcumin concentration increased, the WS of chitosan‐based films decreased. Likewise, Wu et al. (2018) indicated that the WS of films based on gelatin reduced significantly as the curcumin concentration increased.

Water vapor permeability is one of the main property of the packaging system because it changes the quality and shelf life of foods (Abdollahi et al., 2013). The WVP of packaging materials should be as low as possible in order to preserve the quality of foods over storage period (Zhou et al., 2009). The effect of curcumin incorporation on the water barrier of LISG films is given in Table 1. As expected, an increase in curcumin concentration was accompanied by a decrease in WVP. This effect is associated with the hydrophobic nature of curcumin that forms a layer of water vapor molecule barrier. Curcumin is composed of long carbon chain and benzene ring that prolongs the diffusion of gasses molecules through the films (Liu et al., 2018). Additionally, LISG has both hydroxyl and carboxyl groups, and therefore, it can easily interact with water molecules (Fathi et al., 2018). The hydrogen bonding between curcumin and functional groups present in LISG structure decreases the number of polar groups and thus decreases the water permeability of the films (Salarbashi et al., 2017). Moreover, according to FESEM analysis, in the LISG/curcumin films, the entire surface of the films covered by micrometric curcumin crystals which can act as an obstacle for diffusion of water vapor molecules. Decreased WVP in films with the addition of curcumin has been documented in previous researches (Liu et al., 2016; Rostami & Esfahani, 2019; Roy & Rhim, 2020; Wang et al., 2019). Conversely, Pereira and Andrade (2017) demonstrated the incorporation of curcumin into chitosan‐based film increased the value of WVP. In conclusion, since LISG/curcumin films had low level of WVP and water solubility, they can be used as a good candidate for food packaging applications.

#### 3.1.3 Mechanical characteristics

The mechanical performance of the developed films was evaluated by two mechanical parameters including tensile strength (TS) and elongation at break (EB). The results are presented in Table 2. With increasing curcumin concentration from 0% to 0.2%, no significant change was observed in TS and EB, but beyond this point, TS increased significantly. The mechanical performance of films is mainly dependent on the microstructure of films and intermolecular force (Shahbazi, 2017). Therefore, the observed effect may be attributed to the strong interaction between the curcumin and polymer structure by H‐bond formation, which could enhance the mechanical performance of the film (Bonilla et al., 2014; Liu et al., 2018). Interestingly, the TS of the developed films was near to LDPE as a synthetic film. On the other hand, as the curcumin concentration decreased, EB showed a decreasing trend. This effect is due to the smaller molecular length of curcumin than LISG, which reduces the deformation of the developed films (Liu et al., 2016). The results are in agreement with those reported in previous studies (Liu et al., 2016; Rostami & Esfahani, 2019). The results of mechanical analysis demonstrated that the mechanical characteristics of LISG film improved significantly with incorporation of curcumin (*p* ˂ .05), which indirectly exhibited good compatibility between curcumin and LISG matrix.

**TABLE 2:** Mechanical properties of LISG films incorporated with various concentrations of curcumin

Curcumin conc (wt. %)	Tensile strength (MPa)	Elongation at break (% )
0	17.11 ± 118^c^	98.14 ± 2.96^a^
0.2	17.31 ± 1.47^c^	81.14 ± 2.02^b^
0.4	21.14 ± 1.25^bc^	69.44 ± 3.93^c^
0.6	23.21 ± 0.97^a^	56.32 ± 1.73^d^

Different letters in the columns indicate a significant difference of P < 0.05.

### 3.2 FT‐IR analysis

**FIGURE 2:**
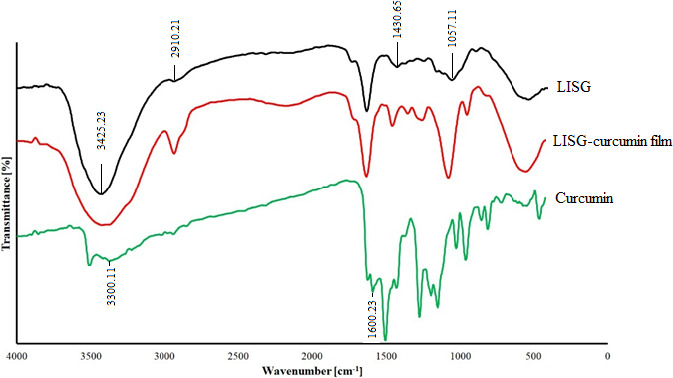
FT‐IR spectra of curcumin, LISG, and LISG/curcumin (0.6%) film

The FT‐IR spectroscopy is commonly used as a useful tool to examine the impact of the additive incorporation on the chemical structure of polymer‐based films by observing the alterations happened in the absorption frequency of the functional groups (Najafi et al., 2020). The FT‐IR spectra of curcumin, neat LISG film, and LISG/curcumin film are presented in Figure 2. In LISG spectrum, the peak located in the wavenumber range of 3,000 to 3,500 cm^‐1^ is assigned to OH groups and the main one around 2,900 cm^‐1^ is associated with the C‐H stretching vibrations of alkane groups in LISG chain (Sinha et al., 2010). The signal at 1,057 cm^‐1^ is related to O‐acetyl groups (Dokht et al., 2018; Percival, 1962). The wavenumber around 1,430 cm^‐1^ is attributed to the symmetrical stretching of carboxylate groups in the uronic acid structure (Vinod et al., 2008). The FT‐IR spectrum of curcumin was also recorded (Figure 2). The peak at 3,300 cm^‐1^ is due to the presence of phenolic hydroxyl groups. The diagnostic peak at 1,600 cm^‐1^ is assigned to the symmetric aromatic ring stretching vibrations (Parveen et al., 2019). In conclusion, it can be seen that there was no considerable change in the FT‐IR spectrum of LISG after curcumin incorporation, suggesting the formation of physical interaction between LISG and curcumin.

### 3.3 Release rate of curcumin

**FIGURE 3:**
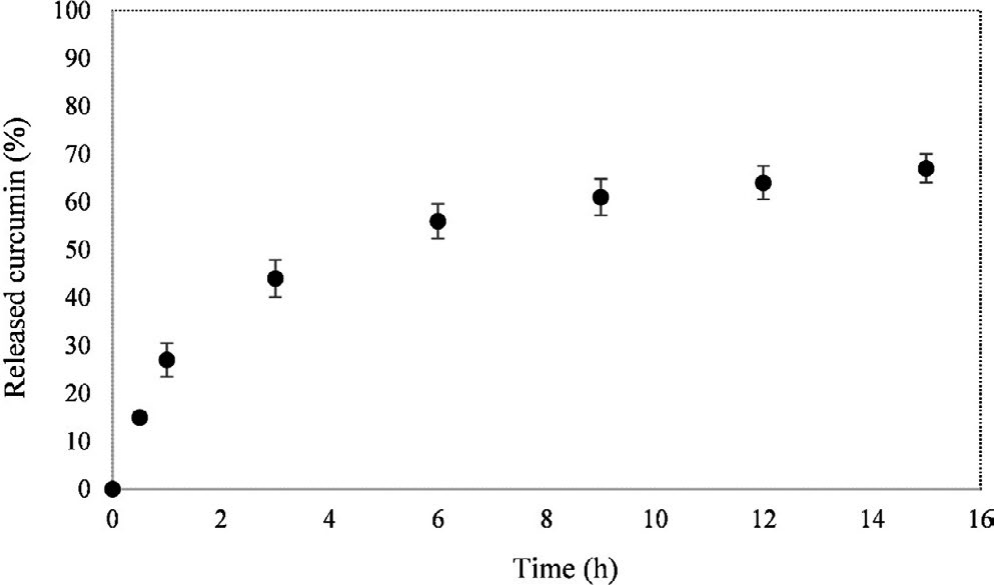
Release profile of curcumin from LISG/curcumin film

The release profile of curcumin in ethanol from LISG/curcumin film is presented in Figure 3. When the contact time with ethanol increased from 0 to 6 hr, the curcumin released rapidly which may be due to the release of curcumin present on the film's surface. The release rate reached equilibrium after 9 hr immersion. LISG/curcumin film can be used as a promising packaging system because the curcumin released at a low rate that leads to restricting the bacterial growth for a prolonged time and preventing oxidation of food products over storage period.

The values of coefficient of determination (*R* ^2^) and root mean square error (RMSE) for the models used to describe the release behavior of curcumin from LISG/curcumin films are given in Table 3. It can be observed that Peppas model was the best model to fit control release data. It has been reported that n parameter in Korsmeyer's Peppas equation is measure of the transport mechanism of bioactive compounds, where *n* ˂ 0.43 shows Fickian diffusion, 0.44 ˂ *n* ˂ 0.85 demonstrates Anomalous transport and *n* ˃ 0.85 reveals Case‐II transport (Siepmann, & Peppas, 2012). As presented in Table 3, the *n* value is <0.43. Therefore, the release of curcumin from LISG/curcumin film in ethanol obeyed Fickian diffusion.

**TABLE 3:** Modeling of curcumin release behavior of LISG/Curcumin films in ethanol solution as a food simulant

Model	*R* ^2^	Adj‐*R* ^2^	RMSE	*k*	*n*
Zero order	.83	.83	2.80	0.17	–
Higuchi model	.97	.99	0.36	1.9	–
Korsmeyer's Peppas model	.99	.99	0.29	2.0	0.41

### 3.4 Antimicrobial, antimold, and antioxidant activity of LISG/curcumin film

Antibacterial and antimold effects of the curcumin incorporated film against the tested bacteria and mold were investigated. The film containing curcumin indicated clear microbial inhibition zones against both gram‐positive and gram‐negative bacteria. The diameters of inhibition zone were in the range of 18–20 mm for the tested bacteria. Additionally, clear antimold activity was observed by in vitro tests (inhibition zone of 13.66 ± 1.59 mm). The H‐bonding and hydrophobic interactions of phenolic compounds such as curcumin and membranal proteins of the bacterial cells change the membranes permeability, consequently, inhibit the bacterial growth (Salgado et al., 2012). Likewise, Manna et al. (2015) demonstrated that curcumin loaded carboxymethylated guar gum could effectively inhibit the growth of both gram‐positive and gram‐negative bacteria. Bajpai et al. (2015) also observed that the chitosan/curcumin‐based films had antibacterial and antimold activity. Conversely, Musso et al. (2017) indicated that the gelatin film loaded by curcumin (0.02% w/v) had no antimicrobial effect against E. *coli*, *S. enteritidis*, *B. cereus*, and *S. aureus*. Since apples and pears are commonly spoiled by *Penicillium expansum*, the developed film can be suggested as a promising packaging for these fruits. However, further experiments should be carried out before use of such packaging in the food and agriculture industry.

The antioxidant activity of neat film and the film incorporated by curcumin was tested by determining DPPH radicals scavenging. The films with and without curcumin showed 5.0 and 43.2% inhibition activity against DPPH radicals. The antiradical activity of neat film is due to the interaction of LISG with free‐radical which resulted in produces the products with high stability (Salehi et al., 2019). The electron‐donating of polysaccharides has been reported in the literature (Malsawmtluangi et al., 2014; Pu et al., 2016; Salehi et al., 2019; Thanzami et al., 2015). The antioxidant activity of LISG/curcumin film was significantly more than neat film (*p* ˂ .05). The antioxidant activity of curcumin has been related to the existence of the ortho‐methoxy groups of curcumin. Overall, the developed film can be introduced as a good candidate for packaging of the foods sensitive to oxidation.

### 3.5 Film response to pH changes

**FIGURE 4:**
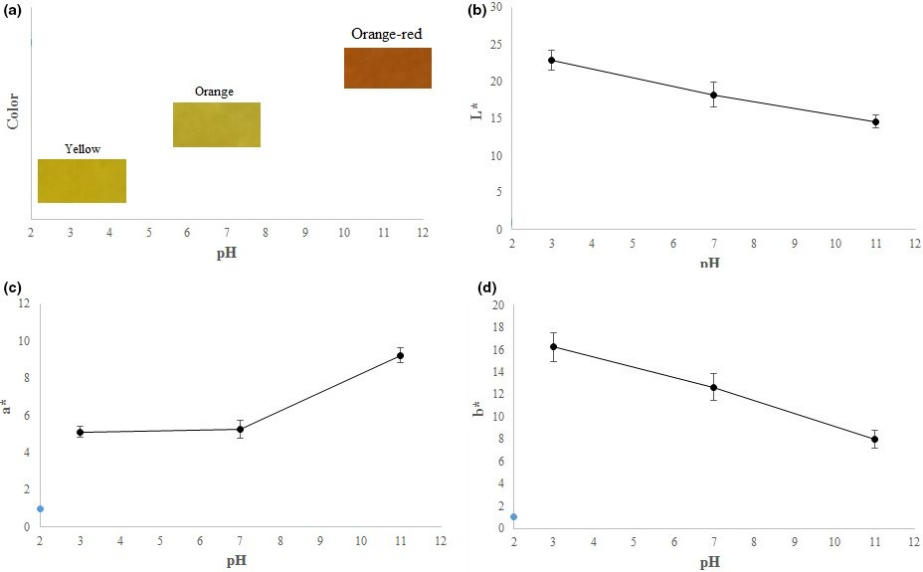
The response of LISG/curcumin film at pH 3, 7, and 11

pH is commonly employed to recognize bacterial growth. To date, several smart packaging systems detecting pH have been fabricated (Kiryukhin et al., 2018; Nopwinyuwong et al., 2010; Pacquit et al., 2007; Rukchon et al., 2014; Salinas et al., 2012). The sensitivity of curcumin to pH change and the presence of carbon dioxide and ammonia have been documented in previous studies (Wannawisan et al., 2018). The response of LISG/curcumin films in contact with acid, neutral, and alkali liquids is depicted in Figure 4a. It can be observed that the developed films had the ability to sense pH changes. Therefore, it can be used to monitor the quality of foods during storage. The color of LISG/curcumin films varied from yellow to orange‐red, in contact with liquids at various pHs. The color change of curcumin in contact with basic medium has been attributed to the deprotonation of some of the functional groups present in curcumin structure at alkaline medium, which increase the polarity of molecule molecules (Rostami & Esfahani, 2019).

The color parameters (L*, a*, and b*) of the films at different pHs are shown in Figure 4b,d. L* value was 22.85 at pH 2 and decreased to 14.65 at pH 11. L* is an indicator of the level of lightness. Thus, with the increase of pH of medium, the color of films becomes darker. An increase in pH of medium from 3 to 7 was accompanied by a slight increase in a* value, but with further increase of pH, the value of a* showed a significant increase, indicating the intensification of the red color. These results are in agreement with those observed in previous studies (Musso et al., 2017; Pereira & Andrade, 2017). Overall, LISG/curcumin films can be employed to measure the pH increase produced by the basic spoilages.

### 3.6 Shrimp spoilage trial

**FIGURE 5:**
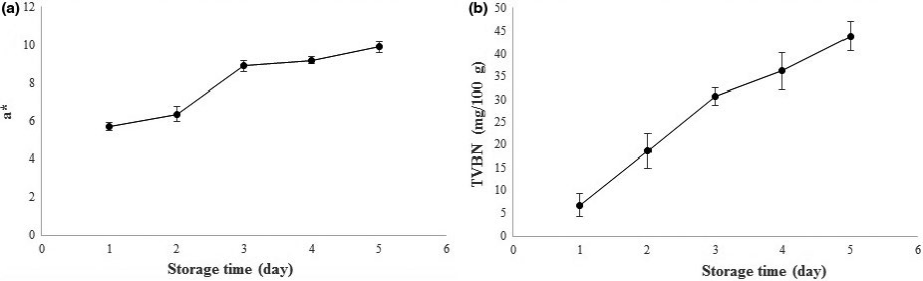
Application of LISG/curcumin film for monitoring shrimp freshness

**FIGURE 6:**
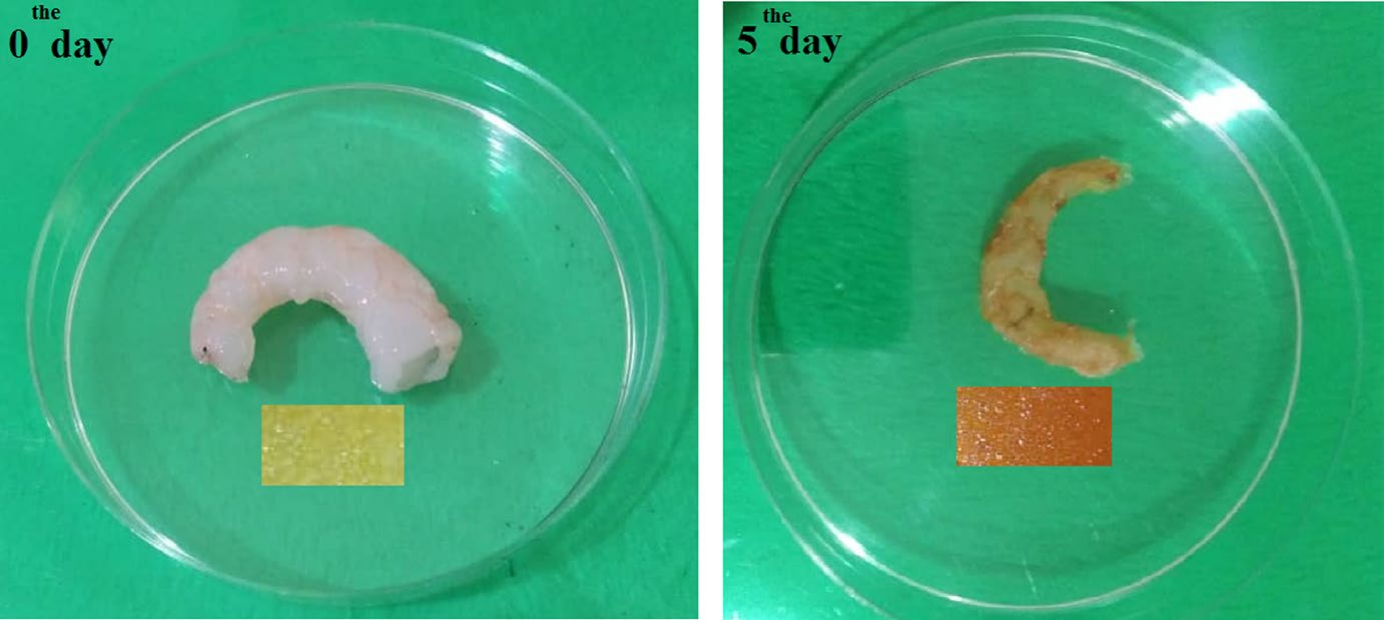
Application of LISG/curcumin film for freshness monitory of shrimp

Microorganisms are the main reason for the spoilage of animal‐based protein foods. Total volatile base nitrogen (TVBN) is commonly used as an indicator for evaluating the seafood spoilage. The production of TVBN reduces the pH of the medium. As mentioned above, LISG/curcumin film had the ability to sense pH changes, and thus the developed film was used as an indicator for monitoring the freshness of shrimp (Figure 5). The value of a* (redness) as a function of storage time is presented in Figure 6. It can be observed that a* value of LISG/curcumin film increased from 5.7 to 9.9 when the storage time increased to 5 days. Correspondingly, the color of the films changed from yellow to red during storage period. During storage period, the TVBN content of the shrimp increased from 6.66 mg 100 g^‐1^ to 43.89 mg 100 g^‐1^, demonstrating the developed smart packaging system could successfully detect the shrimp spoilage. In conclusion, LISG/curcumin film can be introduced as an effective packaging system for sensing the spoilage of seafood such as shrimp. Likewise, Ma et al. (2017) showed that Tara gum/polyvinyl alcohol‐based film could effectively sense the spoilage of shrimp. In another study, Liu et al. (2018) fabricated a smart packaging system based on k‐carrageenan/curcumin to monitor the freshness of pork and shrimp over storage period. They reported that the developed films can be employed as an effective indicator for evaluating the freshness of seafood.

Pearson correlation test was carried out to elucidate whether there was a relationship between the TVBN content and the a*. The results revealed that there is a strong positive correlation between TVBN content of shrimp and the a* values during storage time (Pearson correlation = 0.975).

## 4 CONCLUSION

Tensile strength of the developed films was comparable to synthetic films such as LDPE. The incorporation of curcumin into LISG film improved the WVP of the films. FESEM images demonstrated good compatibility between LISG and curcumin. FT‐IR analysis revealed the formation of physical interaction between LISG and curcumin. The results demonstrated that there is a strong positive correlation between TVBN content of shrimp and the a* during storage time (Pearson correlation = 0.975). LISG/curcumin film exhibited excellent antibacterial/mold and antioxidant activity. Furthermore, it could sense the pH change and thus introduced as a promising packaging to detect spoilage of shrimp.

## CONFLICTS OF INTEREST

We declare that there are no conflict of interest.

## Data Availability

Data available on request due to privacy/ethical restrictions.

## References

[fsn32114-bib-0001] Abdollahi, M. , Alboofetileh, M. , Rezaei, M. , & Behrooz, R. (2013). Comparing physico‐mechanical and thermal properties of alginate nanocomposite films reinforced with organic and/or inorganic nanofillers. Food Hydrocolloids, 32(2), 416–424. 10.1016/j.foodhyd.2013.02.006

[fsn32114-bib-0002] Aliabbasi, N. , Emam‐Djomeh, Z. , & Amighi, F. (2020). Active food packaging with nano/microencapsulated ingredients In Application of nano/microencapsulated ingredients in food products (pp. 171–210). Elsevier.

[fsn32114-bib-0003] ASTM D882‐91 . (1991). Standard test methods for tensile properties of thin plastic In: Annual Book of ASTM. Standards American Society for Testing and Materials. Philadelphia, PA.

[fsn32114-bib-0004] ASTM Standard (1989). Standard test methods for water vapor transmission of materials Annual book of ASTM standards. Designation E96–E80. (pp. 730–739). Philadelphia: ASTM.

[fsn32114-bib-0005] Bajpai, S. , Chand, N. , & Ahuja, S. (2015). Investigation of curcumin release from chitosan/cellulose micro crystals (CMC) antimicrobial films. International Journal of Biological Macromolecules, 79, 440–448. 10.1016/j.ijbiomac.2015.05.012 26003303

[fsn32114-bib-0006] Bonilla, J. , Fortunati, E. , Atarés, L. , Chiralt, A. , & Kenny, J. M. (2014). Physical, structural and antimicrobial properties of poly vinyl alcohol–chitosan biodegradable films. Food Hydrocolloids, 35, 463–470. 10.1016/j.foodhyd.2013.07.002

[fsn32114-bib-0007] Cai, J. , Chen, Q. , Wan, X. , & Zhao, J. (2011). Determination of total volatile basic nitrogen (TVB‐N) content and Warner‐Bratzler shear force (WBSF) in pork using Fourier transform near infrared (FT‐NIR) spectroscopy. Food Chemistry, 126(3), 1354–1360. 10.1016/j.foodchem.2010.11.098

[fsn32114-bib-0008] Dokht, S. K. , Djomeh, Z. E. , Yarmand, M. S. , & Fathi, M. (2018). Extraction, chemical composition, rheological behavior, antioxidant activity and functional properties of Cordia myxa mucilage. International Journal of Biological Macromolecules, 118(Pt A):485‐493. 29909036 10.1016/j.ijbiomac.2018.06.069

[fsn32114-bib-0009] Fabra, M. , Pérez‐Masiá, R. , Talens, P. , & Chiralt, A. (2011). Influence of the homogenization conditions and lipid self‐association on properties of sodium caseinate based films containing oleic and stearic acids. Food Hydrocolloids, 25(5), 1112–1121. 10.1016/j.foodhyd.2010.10.008

[fsn32114-bib-0010] Fathi, M. , Emam‐Djomeh, Z. , & Sadeghi‐Varkani, A. (2018). Extraction, characterization and rheological study of the purified polysaccharide from *Lallemantia ibrica* seeds. International Journal of Biological Macromolecules, 120, 1265–1274. 10.1016/j.ijbiomac.2018.08.159 30170051

[fsn32114-bib-0011] Galus, S. , & Lenart, A. (2013). Development and characterization of composite edible films based on sodium alginate and pectin. Journal of Food Engineering, 115(4), 459–465. 10.1016/j.jfoodeng.2012.03.006

[fsn32114-bib-0012] Gontard, N. , Duchez, C. , Cuq, J.‐L. , & Guilbert, S. (1994). Edible composite films of wheat gluten and lipids: Water vapour permeability and other physical properties. International Journal of Food Science & Technology, 29(1), 39–50. 10.1111/j.1365-2621.1994.tb02045.x

[fsn32114-bib-0013] Govindaraj, P. , Kandasubramanian, B. , & Kodam, K. M. (2014). Molecular interactions and antimicrobial activity of curcumin (*Curcuma longa*) loaded polyacrylonitrile films. Materials Chemistry and Physics, 147(3), 934–941. 10.1016/j.matchemphys.2014.06.040

[fsn32114-bib-0014] Kang, N.‐W. , Kim, M.‐H. , Sohn, S.‐Y. , Kim, K.‐T. , Park, J.‐H. , Lee, S.‐Y. , Lee, J.‐Y. , & Kim, D.‐D. (2018). Curcumin‐loaded lipid‐hybridized cellulose nanofiber film ameliorates imiquimod‐induced psoriasis‐like dermatitis in mice. Biomaterials, 182, 245–258. 10.1016/j.biomaterials.2018.08.030 30142524

[fsn32114-bib-0015] Kiryukhin, M. V. , Lau, H. H. , Goh, S. H. , Teh, C. , Korzh, V. , & Sadovoy, A. (2018). A membrane film sensor with encapsulated fluorescent dyes towards express freshness monitoring of packaged food. Talanta, 182, 187–192. 10.1016/j.talanta.2018.01.085 29501139

[fsn32114-bib-0016] Lai, F. , Wen, Q. , Li, L. , Wu, H. , & Li, X. (2010). Antioxidant activities of water‐soluble polysaccharide extracted from mung bean (*Vigna radiata* L.) hull with ultrasonic assisted treatment. Carbohydrate Polymers, 81(2), 323–329.

[fsn32114-bib-0017] Liu, J. , Wang, H. , Wang, P. , Guo, M. , Jiang, S. , Li, X. , & Jiang, S. (2018). Films based on κ‐carrageenan incorporated with curcumin for freshness monitoring. Food Hydrocolloids, 83, 134–142. 10.1016/j.foodhyd.2018.05.012

[fsn32114-bib-0018] Liu, Y. , Cai, Y. , Jiang, X. , Wu, J. , & Le, X. (2016). Molecular interactions, characterization and antimicrobial activity of curcumin–chitosan blend films. Food Hydrocolloids, 52, 564–572.

[fsn32114-bib-0019] Ma, Q. , Du, L. , & Wang, L. (2017). Tara gum/polyvinyl alcohol‐based colorimetric NH_3_ indicator films incorporating curcumin for intelligent packaging. Sensors and Actuators B: Chemical, 244, 759–766. 10.1016/j.snb.2017.01.035

[fsn32114-bib-0020] Malsawmtluangi, C. , Thanzami, K. , Lalhlenmawia, H. , Selvan, V. , Palanisamy, S. , Kandasamy, R. , & Pachuau, L. (2014). Physicochemical characteristics and antioxidant activity of *Prunus cerasoides* D. Don gum exudates. International Journal of Biological Macromolecules, 69, 192–199. 10.1016/j.ijbiomac.2014.05.050 24875319

[fsn32114-bib-0021] Manna, P. J. , Mitra, T. , Pramanik, N. , Kavitha, V. , Gnanamani, A. , & Kundu, P. (2015). Potential use of curcumin loaded carboxymethylated guar gum grafted gelatin film for biomedical applications. International Journal of Biological Macromolecules, 75, 437–446. 10.1016/j.ijbiomac.2015.01.047 25661877

[fsn32114-bib-0022] Mayet, N. , Kumar, P. , Choonara, Y. E. , Tomar, L. K. , Tyagi, C. , du Toit, L. C. , & Pillay, V. (2014). Synthesis of a semi‐interpenetrating polymer network as a bioactive curcumin film. An Official Journal of the American Association of Pharmaceutical Scientists, 15(6), 1476–1489. 10.1208/s12249-014-0170-3 PMC4245434 24984920

[fsn32114-bib-0023] Musso, Y. S. , Salgado, P. R. , & Mauri, A. N. (2017). Smart edible films based on gelatin and curcumin. Food Hydrocolloids, 66, 8–15. 10.1016/j.foodhyd.2016.11.007

[fsn32114-bib-0024] Najafi, A. , Emam‐Djomeh, Z. , Askari, G. , & Fathi, M. (2020). Electrospun hydrophobe nanofibrous membrane based on polysulfone/Triton x–100: A novel vehicle to concentrate pomegranate juice. Journal of Food Process Engineering, 43(10), e13493 10.1111/jfpe.13493

[fsn32114-bib-0025] Nopwinyuwong, A. , Trevanich, S. , & Suppakul, P. (2010). Development of a novel colorimetric indicator label for monitoring freshness of intermediate‐moisture dessert spoilage. Talanta, 81(3), 1126–1132. 10.1016/j.talanta.2010.02.008 20298903

[fsn32114-bib-0026] Pacquit, A. , Frisby, J. , Diamond, D. , Lau, K. T. , Farrell, A. , Quilty, B. , & Diamond, D. (2007). Development of a smart packaging for the monitoring of fish spoilage. Food Chemistry, 102(2), 466–470. 10.1016/j.foodchem.2006.05.052

[fsn32114-bib-0027] Parveen, S. , Ghosh, P. , Mitra, A. , Gupta, S. , & Dasgupta, S. (2019). Preparation, characterization, and in vitro release study of curcumin‐loaded cataractous eye protein isolate films. Emergent Materials, 2, 475–486. 10.1007/s42247-019-00036-6

[fsn32114-bib-0028] Percival, E. G. V. (1962). Structural carbohydrate chemistry. Structural carbohydrate chemistry (2nd ed.). J. G. Miller.

[fsn32114-bib-0029] Pereira, P. F. , & Andrade, C. T. (2017). Optimized pH‐responsive film based on a eutectic mixture‐plasticized chitosan. Carbohydrate Polymers, 165, 238–246. 10.1016/j.carbpol.2017.02.047 28363546

[fsn32114-bib-0030] Pu, X. , Ma, X. , Liu, L. , Ren, J. , Li, H. , Li, X. , Yu, S. , Zhang, W. , & Fan, W. (2016). Structural characterization and antioxidant activity in vitro of polysaccharides from angelica and astragalus. Carbohydrate Polymers, 137, 154–164. 10.1016/j.carbpol.2015.10.053 26686116

[fsn32114-bib-0031] Robles‐Almazan, M. , Pulido‐Moran, M. , Moreno‐Fernandez, J. , Ramirez‐Tortosa, C. , Rodriguez‐Garcia, C. , Quiles, J. L. , & Ramirez‐Tortosa, M. (2017). Hydroxytyrosol: Bioavailability, toxicity, and clinical applications. Food Research International, 105, 654‐667. 29433260 10.1016/j.foodres.2017.11.053

[fsn32114-bib-0032] Rostami, H. , & Esfahani, A. A. (2019). Development a smart edible nanocomposite based on mucilage of Melissa officinalis seed/montmorillonite (MMT)/curcumin. International Journal of Biological Macromolecules, 141, 171–177. 10.1016/j.ijbiomac.2019.08.261 31479676

[fsn32114-bib-0033] Roy, S. , & Rhim, J.‐W. (2020). Carboxymethyl cellulose‐based antioxidant and antimicrobial active packaging film incorporated with curcumin and zinc oxide. International Journal of Biological Macromolecules, 148, 666–676. 10.1016/j.ijbiomac.2020.01.204 31978467

[fsn32114-bib-0034] Rukchon, C. , Nopwinyuwong, A. , Trevanich, S. , Jinkarn, T. , & Suppakul, P. (2014). Development of a food spoilage indicator for monitoring freshness of skinless chicken breast. Talanta, 130, 547–554. 10.1016/j.talanta.2014.07.048 25159445

[fsn32114-bib-0035] Sadeghi‐Varkani, A. , Emam‐Djomeh, Z. , & Askari, G. (2018a). Morphology and physicochemical properties of a novel *Lallemantia iberica* mucilage/titanium dioxide bio‐nanocomposite. Polymer Testing, 67, 12–21. 10.1016/j.polymertesting.2018.02.006

[fsn32114-bib-0036] Sadeghi‐Varkani, A. , Emam‐Djomeh, Z. , & Askari, G. (2018b). Physicochemical and microstructural properties of a novel edible film synthesized from Balangu seed mucilage. International Journal of Biological Macromolecules, 108, 1110–1119. 10.1016/j.ijbiomac.2017.11.029 29126944

[fsn32114-bib-0037] Salarbashi, D. , Noghabi, M. S. , Bazzaz, B. S. F. , Shahabi‐Ghahfarrokhi, I. , Jafari, B. , & Ahmadi, R. (2017). Eco‐friendly soluble soybean polysaccharide/nanoclay Na+ bionanocomposite: Properties and characterization. Carbohydrate Polymers, 169, 524–532. 10.1016/j.carbpol.2017.04.011 28504176

[fsn32114-bib-0038] Salarbashi, D. , Tafaghodi, M. , Bazzaz, B. S. F. , & Jafari, B. (2018). Characterization of soluble soybean (SSPS) polysaccharide and development of eco‐friendly SSPS/TiO2 nanoparticle bionanocomposites. International Journal of Biological Macromolecules, 112, 852–861. 10.1016/j.ijbiomac.2018.01.182 29410370

[fsn32114-bib-0039] Salehi, E. , Emam‐Djomeh, Z. , Askari, G. , & Fathi, M. (2019). Opuntia ficus indica fruit gum: Extraction, characterization, antioxidant activity and functional properties. Carbohydrate Polymers, 206, 565–572. 10.1016/j.carbpol.2018.11.035 30553358

[fsn32114-bib-0040] Salgado, P. R. , López‐Caballero, M. E. , Gómez‐Guillén, M. C. , Mauri, A. N. , & Montero, M. P. (2012). Exploration of the antioxidant and antimicrobial capacity of two sunflower protein concentrate films with naturally present phenolic compounds. Food Hydrocolloids, 29(2), 374–381. 10.1016/j.foodhyd.2012.03.006

[fsn32114-bib-0041] Salinas, Y. , Ros‐Lis, J. V. , Vivancos, J.‐L. , Martínez‐Máñez, R. , Marcos, M. D. , Aucejo, S. , Herranz, N. , & Lorente, I. (2012). Monitoring of chicken meat freshness by means of a colorimetric sensor array. Analyst, 137(16), 3635–3643. 10.1039/c2an35211g 22768392

[fsn32114-bib-0042] Shahbazi, Y. (2017). The properties of chitosan and gelatin films incorporated with ethanolic red grape seed extract and *Ziziphora clinopodioides* essential oil as biodegradable materials for active food packaging. International Journal of Biological Macromolecules, 99, 746–753. 10.1016/j.ijbiomac.2017.03.065 28315767

[fsn32114-bib-0043] Siepmann, J. , & Peppas, N. A. (2012). Modeling of drug release from delivery systems based on hydroxypropyl methylcellulose (HPMC). Advanced Drug Delivery Reviews, 64, 163–174. 10.1016/j.addr.2012.09.028 11369079

[fsn32114-bib-0044] Sinha, A. , Ghosh, G. , & Roy, M. N. (2010). Conductance and FTIR spectroscopic study of sodium tetraphenylborate in pure 1, 3‐dioxolane and isoamyl alcohol and their binary mixtures. Physics and Chemistry of Liquids, 48(1), 62–78. 10.1080/00319100802654354

[fsn32114-bib-0045] Solghi, S. , Emam‐Djomeh, Z. , Fathi, M. , & Farahani, F. (2020). The encapsulation of curcumin by whey protein: Assessment of the stability and bioactivity. Journal of Food Process Engineering, 43(6), e13403 10.1111/jfpe.13403

[fsn32114-bib-0046] Stuchell, Y. M. , & Krochta, J. M. (1994). Enzymatic treatments and thermal effects on edible soy protein films. Journal of Food Science, 59(6), 1332–1337. 10.1111/j.1365-2621.1994.tb14709.x

[fsn32114-bib-0047] Thakur, G. , Singh, A. , & Singh, I. (2016). Formulation and evaluation of transdermal composite films of chitosan‐montmorillonite for the delivery of curcumin. International Journal of Pharmaceutical Investigation, 6(1), 23 10.4103/2230-973X.176468 27014616 PMC4787059

[fsn32114-bib-0048] Thanzami, K. , Malsawmtluangi, C. , Lalhlenmawia, H. , Seelan, T. V. , Palanisamy, S. , Kandasamy, R. , & Pachuau, L. (2015). Characterization and in vitro antioxidant activity of *Albizia stipulata* Boiv. gum exudates. International Journal of Biological Macromolecules, 80, 231–239. 10.1016/j.ijbiomac.2015.06.043 26118486

[fsn32114-bib-0049] Vinod, V. , Sashidhar, R. , Sarma, V. , & Vijaya Saradhi, U. (2008). Compositional analysis and rheological properties of gum kondagogu (*Cochlospermum gossypium*): A tree gum from India. Journal of Agricultural and Food Chemistry, 56(6), 2199–2207. 10.1021/jf072766p 18318494

[fsn32114-bib-0050] Wang, L. , Xue, J. , & Zhang, Y. (2019). Preparation and characterization of curcumin loaded caseinate/zein nanocomposite film using pH‐driven method. Industrial Crops and Products, 130, 71–80. 10.1016/j.indcrop.2018.12.072

[fsn32114-bib-0051] Wannawisan, N. , Sane, A. , Runglerdkriangkrai, J. , Wilaipun, P. , & Suppakul, P. (2018). Effects of mixed solvent and dye concentration on turmeric immobilized cellophane film potential used as a novel fish spoilage indicator. Food and Applied Bioscience International Conference 2018.

[fsn32114-bib-0052] Wu, J. , Sun, X. , Guo, X. , Ji, M. , Wang, J. , Cheng, C. , Chen, L. , Wen, C. , & Zhang, Q. (2018). Physicochemical, antioxidant, in vitro release, and heat sealing properties of fish gelatin films incorporated with β‐cyclodextrin/curcumin complexes for apple juice preservation. Food and Bioprocess Technology, 11(2), 447–461. 10.1007/s11947-017-2021-1

[fsn32114-bib-0053] Zhou, J. , Wang, S. , & Gunasekaran, S. (2009). Preparation and characterization of whey protein film incorporated with TiO_2_ nanoparticles. Journal of Food Science, 74(7), N50–N56. 19895492 10.1111/j.1750-3841.2009.01270.x

[fsn32114-bib-0054] Zolfi, M. , Khodaiyan, F. , Mousavi, M. , & Hashemi, M. (2014). Development and characterization of the kefiran‐whey protein isolate‐TiO_2_ nanocomposite films. International Journal of Biological Macromolecules, 65, 340–345. 10.1016/j.ijbiomac.2014.01.010 24418333

